# Phosphorylated DegU Manipulates Cell Fate Differentiation in the Bacillus subtilis Biofilm

**DOI:** 10.1128/JB.00930-13

**Published:** 2014-01

**Authors:** Victoria L. Marlow, Michael Porter, Laura Hobley, Taryn B. Kiley, Jason R. Swedlow, Fordyce A. Davidson, Nicola R. Stanley-Wall

**Affiliations:** a Division of Molecular Microbiology, College of Life Sciences, University of Dundee, Dundee, United Kingdom; b Centre for Gene Regulation and Expression, College of Life Sciences, University of Dundee, Dundee, United Kingdom; c Division of Mathematics, University of Dundee, Dundee, United Kingdom

## Abstract

Cell differentiation is ubiquitous and facilitates division of labor and development. Bacteria are capable of multicellular behaviors that benefit the bacterial community as a whole. A striking example of bacterial differentiation occurs throughout the formation of a biofilm. During Bacillus subtilis biofilm formation, a subpopulation of cells differentiates into a specialized population that synthesizes the exopolysaccharide and the TasA amyloid components of the extracellular matrix. The differentiation process is indirectly controlled by the transcription factor Spo0A that facilitates transcription of the *eps* and *tapA* (*tasA*) operons. DegU is a transcription factor involved in regulating biofilm formation. Here, using a combination of genetics and live single-cell cytological techniques, we define the mechanism of biofilm inhibition at high levels of phosphorylated DegU (DegU∼P) by showing that transcription from the *eps* and *tapA* promoter regions is inhibited. Data demonstrating that this is not a direct regulatory event are presented. We demonstrate that DegU∼P controls the frequency with which cells activate transcription from the operons needed for matrix biosynthesis in favor of an off state. Subsequent experimental analysis led us to conclude that DegU∼P functions to increase the level of Spo0A∼P, driving cell fate differentiation toward the terminal developmental process of sporulation.

## INTRODUCTION

Cell differentiation is ubiquitous and facilitates both division of labor and development. Bacterial communities benefit from multicellular behaviors (1, 2), but it remains unknown how bacteria coordinate mutually exclusive cell states within the population. A striking example of cell differentiation in bacteria is the formation of a biofilm (3), a multicellular sessile community of bacteria encased within a self-produced polymeric matrix (4). Understanding the processes that instigate and control biofilm formation is important for the development of methods needed to control chronic infections and promote bioremediation and biocontrol processes (5, 6). Fundamental to understanding phenotypic heterogeneity in the biofilm is a mechanistic knowledge at the molecular level of how bacteria decide between alternate, and often incompatible, cell fates.

Our understanding of how the Gram-positive bacterium Bacillus subtilis integrates environmental and other regulatory signals to coordinate the complex decision-making processes that control biofilm formation has progressed substantially since the first reports of its biofilm-forming capability (7, 8). An essential feature of a biofilm is the production of the extracellular matrix (9). In the B. subtilis biofilm, a subpopulation of the isogenic bacterial community produces two of the three extracellular components that are needed to allow biofilm maturation (10). The *tapA-sipW-tasA* operon (here *tapA*) is responsible for the production of TasA, a secreted protein found in the biofilm matrix that forms amyloid-like fibers that act as structural bridges between cells (11, 12). The products of the 15-gene *epsA-epsO* operon are required for the synthesis of the exopolysaccharide component of the biofilm matrix and the concomitant inhibition of flagellum-mediated motility (7, 13). In addition to the exopolysaccharide and TasA amyloid fibers, a third component needed for biofilm matrix assembly is synthesized by the whole biofilm population and is encoded by the monocistronic *bslA* gene (formerly *yuaB*) (14, 15). BslA is a small extracellular protein that functions synergistically with the TasA amyloid fibers and exopolysaccharide to facilitate the assembly of the biofilm matrix (16). Further analysis has defined BslA to be a self-assembling bacterial hydrophobin that forms a hydrophobic coating on the biofilm (17, 18).

**FIG 1:**
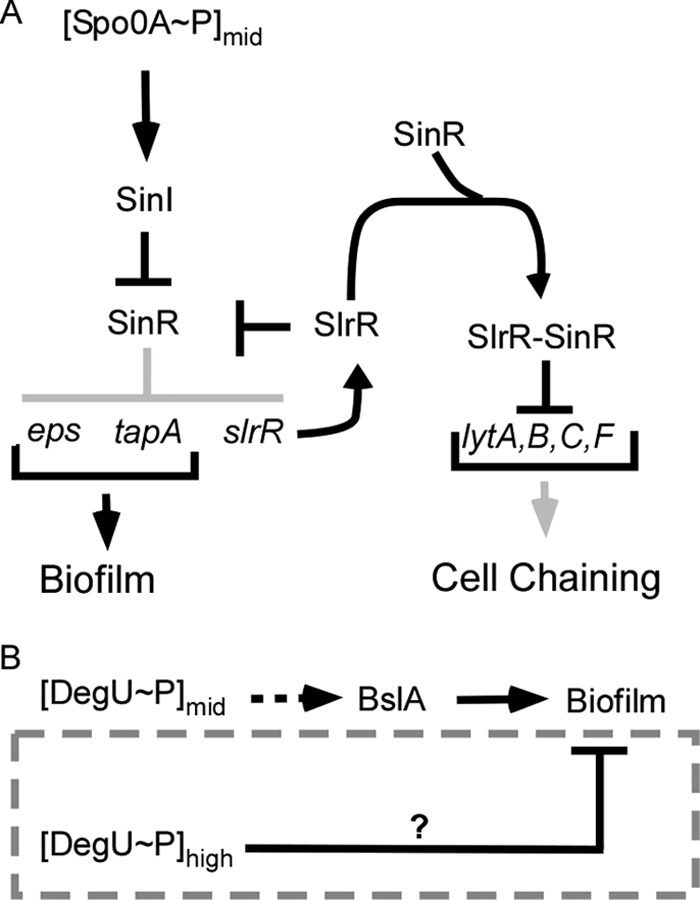
Schematic diagrams indicating the regulatory network controlling biofilm formation. (A) Details of the mechanism used by Spo0A∼P to control biofilm formation and autolysin production. (Adapted from *Genes & Development* [33].) The light gray color represents pathways that are inactive when SpoA∼P is found at intermediate levels in the cell. (B) Schematic representation of the role of DegU∼P in controlling biofilm formation by B. subtilis. The dashed gray box highlights the focus of this study. The dashed arrow represents an indirect regulatory event. The T bar represents inhibition, and arrows represent activation.

Production of the macromolecules that form the biofilm matrix is tightly regulated by a genetic network dependent on the activation of the two main transcription factors, Spo0A and DegU (19–21) (Fig. 1A and B). Transcription from both the *tapA* and *eps* promoters is directly inhibited by the repressor proteins SinR and AbrB (19, 22, 23). Relief of inhibition occurs once a critical, or threshold, level of phosphorylated Spo0A (Spo0A∼P) is reached (24). Five sensor kinases, KinA to KinE, trigger a phosphorelay which culminates in the phosphorylation of Spo0A (25). The sensor kinases are activated in response to various environmental signals (26–30). Once Spo0A∼P levels reach the threshold needed to activate biofilm formation, two parallel pathways of antirepression are invoked and the promoter regions are released (22, 31, 32). More specifically, SinR is sequestered from the promoter elements due to the production of an antirepressor protein called SinI and activation of the SlrR-bistable switch (33). When present, SlrR further sequesters SinR from the *eps* and *tasA* promoter regions, allowing transcription (33). Concomitantly, activation of the SlrR-bistable switch represses transcription of the genes required for the synthesis of autolysins (Fig. 1A) (33), further promoting the switch from a motile lifestyle to a sessile biofilm lifestyle. The activated SlrR-bistable switch state is inherited by daughter cells due to the presence of a self-reinforcing positive-feedback loop and propagates matrix gene expression in the population (33).

Synthesis of BslA is also subject to complex regulatory events. Transcription of *bslA* is directly repressed by AbrB (21) and indirectly activated by the transcription factor Rok (15). The *bslA* gene is the main target (indirectly) activated by intermediate levels of phosphorylated DegU (DegU∼P) during activation of biofilm formation (Fig. 1B) (14). DegU is a response regulator that is phosphorylated by its cognate sensor histidine kinase, DegS (34). A direct consequence of the lack of BslA biosynthesis is that the *degU* and *degSU* mutant strains exhibit biofilm-minus phenotypes (14, 16). Indeed, heterologous expression of *bslA* in the *degU* mutant background is both necessary and sufficient to recover biofilm formation (16). However, the role of DegU∼P during biofilm formation is complex. In addition to functioning as an activator at intermediate levels of DegU∼P (14, 35, 36), it can also function to inhibit biofilm formation when the levels increase beyond an upper threshold level (36) (Fig. 1B).

**FIG 2:**
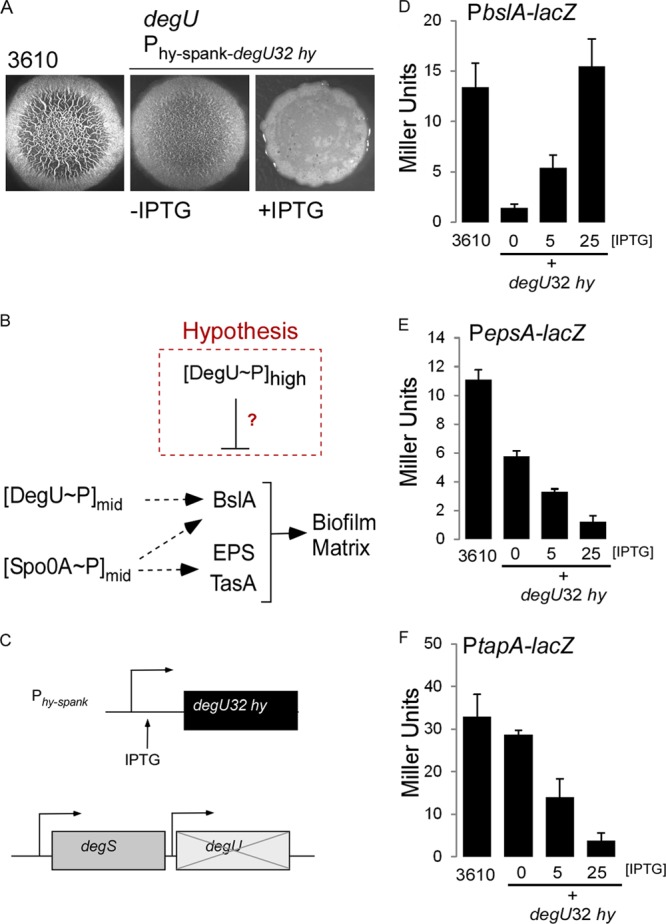
DegU∼P inhibits transcription from the *epsA* and *tapA* promoter regions. (A) Biofilm morphology indicated by the formation of a complex colony on MSgg agar after 24 h of incubation at 37°C for the wild-type strain NCIB3610 (3610) and strain *degU P*(Hy)-*spank-degU32*(Hy)-*lacI* (NRS1325); 25 μM IPTG was used to induce transcription in strain NRS1325, as indicated. (B) Schematic representation of the role of DegU∼P and Spo0A∼P in controlling biofilm formation by B. subtilis. The broken red box highlights our hypothesis that high levels of DegU∼P inhibit transcription from the matrix operons. (C) Schematic representation of the *degU32*(Hy) strain (NRS1325) used in the analysis. (D) β-Galactosidase activity generated from the *PbslA-lacZ* reporter fusion in wild-type strain 3610 (NRS2052) and the *degU*32(Hy) strain [*degU P*(Hy)-*spank-degU32*(Hy)-*lacI* (NRS2226)]. (E) As for panel D, but data are shown for the *PepsA-lacZ* reporter fusion in the wild-type strain 3610 (NRS1529) and the *degU*32(Hy) strain [*degU P*(Hy)-*spank-degU32*(Hy)-*lacI* (NRS1553)]. (F) As for panel D, but data are shown for the *PtapA-lacZ* reporter fusion in wild-type strain 3610 (NRS1503) and the *degU*32(Hy) strain [*degU P*(Hy)-*spank-degU32*(Hy)-*lacI* (NRS1515)]. Data presented in panels D to F are averages from 3 experiments, where the error bars represent the standard error of the mean. IPTG was added as indicated.

We are interested in how B. subtilis controls and integrates multiple cell fates in its population. Here we focus on the mechanism underpinning our previous observation that high levels of DegU∼P inhibit biofilm formation (36) (Fig. 2A). Here, we employ a combination of genetics, flow cytometry, and real-time single-cell fluorescence microscopy to demonstrate that DegU∼P inhibits transcription from both the *epsA* and *tapA* promoter regions, thus explaining the block in biofilm formation at high levels of DegU∼P. We show that this novel role of DegU∼P is physiologically relevant, as in the wild-type biofilm, DegU∼P controls the frequency of cells activating transcription of the genes needed for the biosynthesis of the biofilm matrix. The *in vitro* and *in vivo* data that we present here indicate that this is not a consequence of a direct regulatory event but point toward DegU∼P indirectly manipulating cell fate in favor of a matrix off state. We provide experimental evidence demonstrating that increases in DegU∼P levels drive cell fate differentiation toward the terminal developmental process of sporulation, a developmental process associated with high levels of Spo0A∼P. We conclude that increased levels of DegU∼P result in increased levels of Spo0A∼P.

## MATERIALS AND METHODS

### General strain construction and growth conditions.

The B. subtilis strains used and constructed in this study are described in detail in Table S1 in the supplemental material. Escherichia coli strain MC1061 [F′ *lacIQ lacZM15* Tn*10* (*tet*)] was used for the construction and maintenance of plasmids. B. subtilis JH642 and 168 derivatives were generated by transformation of genetically competent cells with plasmids or DNA using standard protocols (37). SPP1 phage transductions, for introduction of DNA into B. subtilis strain NCIB3610 (here strain 3610), were performed as described previously (36, 38). Both E. coli and B. subtilis strains were routinely grown in Luria-Bertani (LB) medium (10 g NaCl, 5 g yeast extract, 10 g tryptone per liter) or MSgg medium (5 mM potassium phosphate and 100 mM MOPS [morpholinepropanesulfonic acid] at pH 7.0 supplemented with 2 mM MgCl_2_, 700 μM CaCl_2_, 50 μM MnCl_2_, 50 μM FeCl_3_, 1 μM ZnCl_2_, 2 μM thiamine, 0.5% glycerol, 0.5% glutamate) (7) at 37°C or 30°C. When appropriate, antibiotics were used as required at the following concentrations: ampicillin, 100 μg ml^−1^; chloramphenicol, 5 μg ml^−1^; erythromycin, 1 μg ml^−1^, with lincomycin, 25 μg ml^−1^; kanamycin, 25 μg ml^−1^; and spectinomycin, 100 μg ml^−1^. When required IPTG (isopropyl-β-d-thiogalactopyranoside) was added to the medium at the concentrations specified below.

### Biofilm formation assays.

Analysis of biofilm formation was performed as previously described (7, 36). Briefly, 10 μl of a mid-exponential-phase culture grown in LB medium was spotted onto an MSgg agar plate and incubated at 37°C for the time period indicated.

### Flow cytometry.

The fluorescence of strains harboring *gfp* and *yfp* promoter fusions was measured in single cells extracted under biofilm-forming conditions after incubation at either 30°C or 37°C for the times defined as previously described (3, 22).

### β-Galactoside assays.

The β-galactosidase activity of strains harboring *lacZ* promoter reporter fusions was measured as previously described (21, 36). (When required, IPTG was added as indicated below.) The values presented are the average β-galactosidase activities (in Miller units) (39) determined from at least three independent samples. The standard errors of the means are indicated by the bars in the appropriate figures.

### Luciferase assays.

Complex colonies harboring the *PsdpA-lux* fusion were collected as described previously for flow cytometry (3, 22), with the exception that the cells were not fixed but were washed once in 1× phosphate-buffered saline (PBS). Following this, the cells were then subjected to gentle sonication (so that the cells did not lyse [11]). Prior to measurement, the samples were normalized to an optical density at 600 nm of 0.1. Luminescence was measured in 96-well plates (Nunc MicroWell white polystyrene optical-bottom plates) using a MicroLumat Plus luminometer (EG&G Berthold, Regensdorf, Switzerland). The values presented are the average luminescence values normalized against the cell density determined from three biological replicates. The standard errors of the means are indicated by the bars in the appropriate figures.

### Electrophoretic mobility shift assay (EMSA).

A PCR product corresponding to the promoter region of *epsA* was amplified using primers NSW52 (5′-TGG AGA ATT CTG TAC GGC TTG CAC TAA ATG TAC G-3′) and NSW53 (5′-GCC AGA ATT CGG ATC CAT TCA TAG CCT TCA GCC TTC CCG-3′), and a PCR product corresponding to the promoter region of *tapA* was amplified using primers NSW50 (5′-TGG CGA ATT CAT AGA CAA ATC ACA CAT TGT TTG ATC A-3′) and NSW51 (5′-GCC AGA ATT CGG ATC CAT CTT ACC TCC TGT AAA ACA CTG TAA-3′). The products were purified by gel extraction. As a positive control, the promoter region of *aprE* was amplified using primers NSW61 (5′-GGTAAAGCCTATGAATTCTCCATTTTCTTC-3′) and NSW654 (5′-GTCTAAGCTTGATCCACAATTTTTTGCT-3′). The promoter DNA was labeled using 50 μCi [γ-^32^P]ATP (Perkin-Elmer) and T4 polynucleotide kinase (New England BioLabs). Unincorporated ATP was removed from the labeled DNA using an Illustra Microspin G-25 column (GE Healthcare). Phosphorylated purified DegU was produced as described previously (40), with the exception that 15 μM purified DegU∼P and 3.18 μM purified DegS (final concentrations) were added to the phosphorylation reaction mixture. The phosphorylation reaction, DNA binding, and mobility shift assay were performed as described previously (36, 40).

### Time-lapse microscopy.

Single colonies of B. subtilis were inoculated into 5 ml of MSgg medium and grown overnight at 30°C and 220 rpm. The next morning, cells were diluted 25-fold into 3 ml of 15% MSgg medium. After approximately 4 h of incubation at 30°C and 220 rpm or when the cells had reached mid-exponential phase of growth, they were diluted to an optical density at 600 nm (OD_600_) of 0.007 in fresh 15% MSgg medium. This enabled the visualization of single cells with the appropriate spacing for the start of the time-lapse acquisition. Two microliters of this cell suspension was inoculated onto a thin matrix of 15% MSgg medium supplemented with 1.5% agarose (Invitrogen ultrapure agarose) on a microscope slide. Each slide was prepared as follows. A 125-μl Gene Frame (AB-0578; ABgene House, Epsom, Surrey, United Kingdom) was attached to a standard microscope slide (VWR superpremium). The Gene Frame was next filled with molten 15% MSgg medium supplemented with 1.5% agarose (15% MSgg–agarose), IPTG was added at the defined concentrations, where appropriate, and the Gene Frame was covered firmly with a standard microscope slide, to flatten the agarose surface. When the 15% MSgg–agarose had sufficiently cooled and solidified, the upper slide was carefully removed and the 15% MSgg–agarose was carefully removed with a surgical scalpel blade (Swann Morton number 11), leaving behind either one or two strips of MSgg-agarose (∼1.5 mm wide) in the center of the Gene Frame. For experiments where two or more strips were required, the strips were spaced at least 4 mm apart, since previous work (41) has established that these conditions provide air cavities that are essential for efficient growth of B. subtilis. After inoculation, the cell suspension was allowed to dry, after which the Gene Frame was sealed with a number 1.5 coverslip (22 by 22 mm; VWR). The microscope slides were incubated at 30°C in a temperature-controlled environmental chamber (Weather Station; Applied Precision). Prior to the start of acquisition, the cells were allowed to equilibrate on the agarose pads for 3 h. Time-lapse imaging of microcolony development and *PtapA-gfp* expression was performed using a DeltaVision Core wide-field microscope (Applied Precision) mounted on an Olympus IX71 inverted stand with an Olympus ×60 (numerical aperture [NA], 1.4) lens and CoolSNAPHQ camera (Photometrics) with differential interference contrast (DIC) and fluorescence optics. For each experiment 12 independent fields, each containing one or two cells, were manually identified, and their *xyz* positions were stored in the microscope control software (softWoRx; Applied Precision). Data sets (512 by 512 pixels with 2-by-2 binning and 12 *z* sections spaced by 1 μm) were acquired every 15 min for up to 12 h. Green fluorescent protein (GFP) was imaged using a 100-W Mercury lamp and a fluorescein isothiocyanate (FITC) filter set (excitation [EX] wavelength, 490/20 nm; emission [EM] wavelength, 528/38 nm) with an exposure time of 50 ms. DIC images were acquired with a light-emitting diode (LED)-transmitted light source (Applied Precision) at 32% intensity and exposure times of between 25 and 50 ms. Postacquisition data sets were rendered and analyzed using OMERO software (http://openmicroscopy.org). Cell lineage tracking over time was performed manually, while cell length measurements and fluorescence intensity analyses were performed in OMERO software. Cell genealogy trees were generated to scale in Canvas (version 11) software from the manually collated data, and movies were generated using softWoRx (Applied Precision). The threshold used to define activation of the transcriptional reporter *PtapA* was set as a GFP fluorescence intensity value greater than 2 standard deviations above the mean background fluorescence.

Real-time microscopy assessing *PsspB-yfp* expression under conditions of high DegU∼P was also performed as described above, with the exception that yellow fluorescent protein (YFP) was imaged using a 100-W mercury lamp and an FITC filter set (EX wavelength, 490/20 nm; EM wavelength, 528/38 nm) with an exposure time of 300 ms.

### Microscopy of cells harvested from complex colonies. (i) DIC of cells carrying the *PsspB-yfp* fusion.

Colony biofilms were grown as described before (7, 36) and harvested as previously described for flow cytometry (3, 22). Two microliters of the cell suspension was spotted onto a 1.5% agarose pad, and images were acquired using a DeltaVision Core wide-field microscope (Applied Precision) mounted on an Olympus IX71 inverted stand with an Olympus ×100 (NA, 1.4) lens and Cascade2 512 electron-multiplying charge-coupled device (EMCCD) camera (Photometrics). Data sets (512 by 512 pixels with 13 *z* sections spaced by 0.2 μm) were acquired with DIC and fluorescence optics. YFP was imaged using a 100-W mercury lamp and an FITC filter set (EX wavelength, 490/20 nm; EM wavelength, 528/38 nm) with an exposure time of 100 ms. DIC images were acquired with an LED-transmitted light source (Applied precision) at 32% intensity and exposure times between 25 and 50 ms. Postacquisition data sets were rendered and analyzed using OMERO software (http://openmicroscopy.org).

### (ii) Phase-contrast microscopy.

Colony biofilms were grown as described before (7, 36) and harvested as previously described for flow cytometry (3, 22), with the exception that the cells were not fixed. After washing the cells in 1× PBS, the cells were diluted 10-fold into GTE buffer (50 mM glucose, 10 mM EDTA at pH 8, 20 mM Tris-HCl at pH 8) and imaged using a ×100 Plan-Neofluar 1.30 oil immersion lens on an Axio Imager M1 microscope mounted with an Axiocam MRm camera (Zeiss). Images were analyzed using AxioVision, release 4.8, software.

### Statistical analysis.

For details of the statistical analyses used, refer to the supplemental material.

## RESULTS

### High DegU∼P levels reduce expression from the *eps* and *tasA* promoters but not the *bslA* promoter.

High levels of DegU∼P inhibit biofilm formation (Fig. 1B and 2A) (36). We hypothesized that this could be due to inhibition of transcription from the *epsA*, *tapA* (which controls production of TasA), or *bslA* (formerly *yuaB*) coding region (Fig. 2B), preventing the synthesis of the biofilm matrix. To isolate the impact of DegU∼P, a strain was used where the native copy of *degU* was deleted and replaced with the *degU32*(Hy) gene under the control of an IPTG-inducible promoter [*P*(Hy)-*spank*] at the nonessential *amyE* locus (Fig. 2C). The *degU32*(Hy) gene encodes a mutant allele of DegU where the histidine at position 12 is replaced with a leucine (42). The DegU32(Hy) protein has a reduced ability to be dephosphorylated (42), which results in an increase in the level of DegU∼P in the cell by comparison with the level achieved when the wild-type allele of *degU* is induced (36). Into this strain, a *PbslA-lacZ*, *PepsA-lacZ*, or *PtapA-lacZ* reporter fusion was introduced (see Table S1 in the supplemental material). Transcription from the reporter was measured in cells grown in MSgg medium under liquid culture conditions in the presence of IPTG, as indicated below. Previously published data have shown that DegU activates transcription of *bslA* (14, 16), and consistent with this, it was observed that in the absence of induction of *degU32*(Hy), the level of *bslA* transcription was low (Fig. 2D). However, when the *degU32*(Hy) gene was induced by the addition of IPTG, an increased level of transcription from the *bslA* promoter region was apparent (Fig. 2D). These findings indicate that transcription from the *bslA* promoter is activated, and not inhibited, in the presence of high levels of DegU∼P. Note that it has previously been demonstrated that regulation of *bslA* transcription by DegU∼P is not directly mediated (16). In contrast to *bslA* expression, transcription from both the *epsA* and *tapA* promoter regions significantly decreased when the *degU32*(Hy) gene was induced by the addition of IPTG (Fig. 2E and F, respectively). These findings demonstrate that transcription from the *epsA and tapA* promoter elements is inhibited in the presence of high levels of DegU∼P.

### High levels of DegU∼P reduce the number of cells that activate transcription of the biofilm matrix operons.

**FIG 3:**
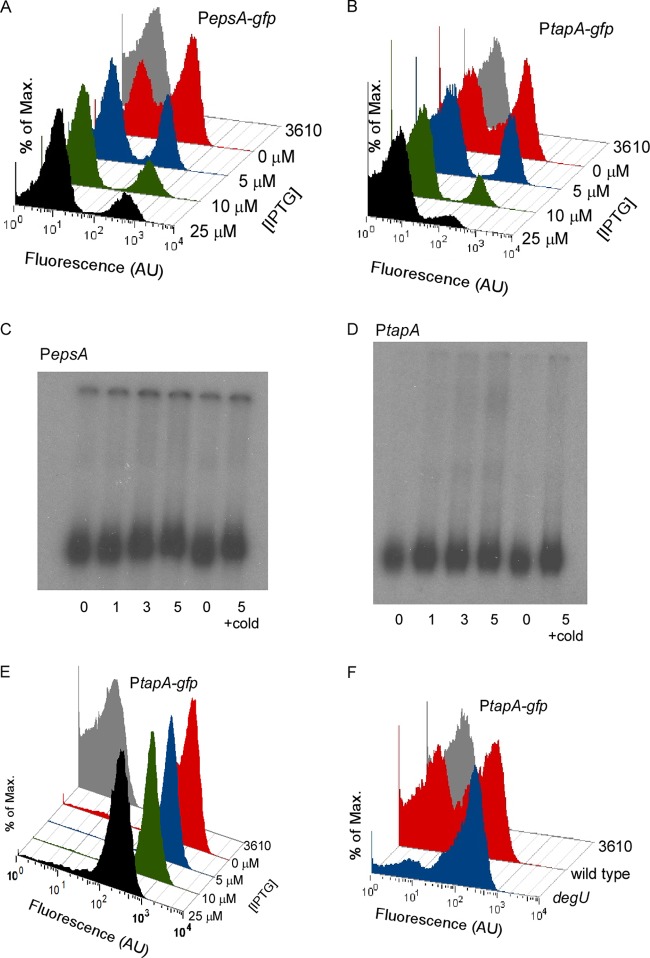
DegU∼P indirectly controls matrix gene expression. (A) Flow cytometry analysis of NRS2245 [3610 *sacA*::*PepsA-gfp degU P*(Hy)-*spank-degU32*(Hy)-*lacI*] cells grown under biofilm formation conditions for 17 h. Cells were grown in the presence or absence of IPTG, as indicated. (B) As for panel A, but data are shown for strain NRS2759 [3610 *sacA*::*PtapA-gfp degU* *P*(Hy)-*spank-degU32*(Hy)-*lacI*]. (C) Electrophoretic mobility shift analysis of the *epsA* operon promoter region. DNA binding reactions were conducted with γ-^32^P-labeled DNA. One nanogram labeled DNA was loaded into each lane, with or without purified DegU∼P-_His6_ at the concentration indicated (μM). (D) As described for panel C but for the *PtapA* promoter region. (E) Flow cytometry analysis of cells grown under biofilm formation conditions for 17 h for strain NRS3424 [3610 *sinR* *P*(Hy)-*spank-degU32*(Hy)-*lacI* *degU* *sacA*::*PtapA-gfp*]. Cells were grown in the presence or absence of IPTG, as indicated. (F) Flow cytometry analysis of NRS2394 (3610 *sacA*::*PtapA-gfp*) and NRS2745 (*degU* *sacA*::*PtapA-gfp*) cells grown under biofilm formation conditions for 17 h. For all flow cytometry histograms shown, strain 3610 was used as a nonfluorescent control. Fluorescence on the *x* axis is in arbitrary fluorescent units (AU), with each data set representative of the trends observed in three independent experiments.

As transcription from the *epsA* and *tapA* promoter regions is bimodal (3, 10), we performed flow cytometry analyses to determine if (i) the number of cells activating transcription was reduced when the level of DegU∼P was increased or (ii) the level of expression on a per cell basis was reduced. Either mechanism would result in a decrease in expression at the population level (Fig. 2E and F). To distinguish between these possibilities, cells carrying either the *PepsA-gfp* (NRS2245) or *PtapA-gfp* (NRS2759) reporter fusion in the *degU32*(Hy) gene background were isolated from complex colonies as previously described (3, 22). Flow cytometry analysis revealed that the cells displayed a clear bimodal transcription profile when grown in the absence of IPTG (Fig. 3A and B, respectively) (10). For simplicity, the two populations are referred to here as matrix off (GFP negative) and matrix on (GFP positive). When the level of IPTG in the growth medium increased and, thus, the level of DegU∼P increased, the proportion of matrix on cells decreased (Fig. 3A and B). Analysis of the *PepsA-gfp* reporter fusion indicated that the percentage of matrix on cells decreased from 58% in the absence of IPTG to 19% in the presence of 25 μM IPTG (Fig. 3A). A similar decrease in the number of matrix on cells was observed for the *PtapA-gfp* reporter fusion, where 40% of cells were matrix on in the absence of IPTG and 8% were matrix on in the presence of 25 μM IPTG (Fig. 3B). These findings demonstrate that the presence of high levels of DegU∼P reduces the number of cells activating transcription from both the *epsA* and *tapA* promoter regions. These data reveal for the first time why biofilm formation is inhibited when the level of DegU∼P is high.

### DegU∼P does not directly regulate matrix gene expression.

To establish whether DegU∼P directly regulates transcription from the *epsA* or *tapA* promoter regions, an EMSA was performed. DegU labeled with His6 (DegU-_His6_) was purified and phosphorylated *in vitro* using purified DegS-_His6_ (36, 40). Prior to EMSA analysis, we confirmed that the purified DegS-_His6_ was capable of phosphorylating DegU-_His6_ *in vitro* using [γ-^32^P]ATP (see Fig. S1A in the supplemental material). No interaction between DegU∼P and either the *epsA* or *tapA* promoter region was observed (Fig. 3C and D, respectively). These experiments were also conducted using purified and phosphorylated DegU32(Hy) protein, and likewise, no interaction with the promoter regions was observed (data not shown). In contrast, a positive-control experiment with a known directly regulated target of DegU∼P resulted in a band shift (the *aprE* promoter region) 43, 44) (see Fig. S1B in the supplemental material).

As the results from the EMSA analysis indicated a lack of direct interaction and, in principle, could be due to a problem with the function of the protein *in vitro*, an *in vivo* genetic approach was also taken to address if DegU∼P interacted directly with the *tapA* promoter region. To do this, the gene encoding the transcriptional repressor SinR was deleted from the chromosome of the strain carrying the *PtapA-gfp* reporter in the *degU32*(Hy) expression strain. Deletion of *sinR* allows constitutive expression from the *tapA* promoter region (Fig. 1A) (23) and resulted in a unimodal matrix on population (Fig. 3E). Production of DegU_H12L_∼P in the *sinR* deletion strain background did not inhibit transcription from the *PtapA-gfp* reporter (Fig. 3E). These results indicate that DegU∼P acts on an upstream step of the regulatory pathway that controls *tapA* gene expression. Therefore, taken together, these data demonstrate that DegU∼P indirectly regulates transcription from the *epsA* and *tapA* promoter regions.

### DegU∼P controls transcription of the *tapA* operon in the wild-type biofilm.

The analyses reported above assessed the impact of DegU∼P on matrix gene expression using a strain where the level of DegU∼P in the cell was controlled using an artificial promoter. Therefore, we next determined the impact that native (physiological) levels of DegU∼P had on cell fate differentiation in the wild-type biofilm. To do this, we deleted *degU* from the chromosome and assessed matrix gene expression using the *PtapA-gfp* reporter fusion (see Table S1 in the supplemental material). We predicted that deletion of *degU* would increase the proportion of matrix on cells in the population by comparison with the proportion for the wild-type strain. The percentage of cells that had initiated transcription was calculated using flow cytometry. Consistent with DegU∼P inhibiting transcription from the *tapA* promoter in the wild-type strain, deletion of *degU* increased the proportion of matrix on cells in the population (Fig. 3F). An increase from 42% matrix on cells in the wild type to 72% matrix on cells in the *degU* mutant was observed (Fig. 3F). As expected, DegU∼P was specifically responsible for modulating expression from the *PtapA* promoter; complementation could be achieved only when the wild-type *degU* coding region was used and not when an allele of *degU* carrying a mutation in the aspartic acid (D56N) residue (and therefore incapable of being phosphorylated by DegS during signal transduction) was used (see Fig. S2 in the supplemental material). These data demonstrate that DegU∼P controls matrix gene expression in the wild-type B. subtilis biofilm. It should be noted that despite an increase in the number of cells in the *degU* population that activated transcription from the *eps* and *tapA* operons, the mature biofilm did not develop. This was due to a lack of transcription from the *bslA* gene, as previously described (14, 16).

### Recovering biofilm matrix assembly does not restore balance to the matrix bimodal population.

**FIG 4:**
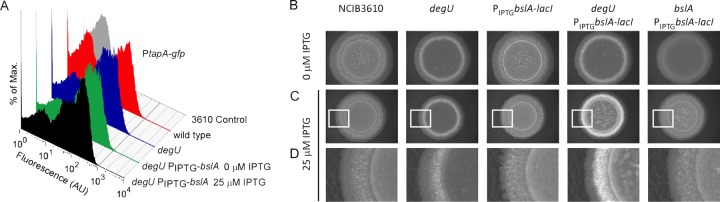
The *degU* mutant biofilm architecture does not influence matrix gene expression. (A) Flow cytometry analysis of cells grown under biofilm formation conditions for 17 h for strains NRS2394 (3610 *sacA-PtapA-gfp*), NRS2745 (*degU sacA-PtapA-gfp*), and NRS3067 (*degU sacA-PtapA-gfp amyE-P*_IPTG_-*bslA-lacI*) in the presence or absence of IPTG, as indicated. Strain 3610 was used as a nonfluorescent control. Fluorescence on the *x* axis is in arbitrary fluorescent units (AU). The data sets are representative of the results observed in three independent experiments. (B to D) Biofilm morphology, as indicated by the formation of a complex colony on MSgg agar after 17 h, for wild-type strain NCIB3610 (3610), a *degU* strain (NRS1314), NCIB3610 carrying *amyE-P*_IPTG_-*bslA-lacI* (NRS2297), *degU amyE-P*_IPTG_-*bslA-lacI*) (NRS2298), and *bslA amyE-P*_IPTG_-*bslA-lacI* (NRS2299). (B) 0 μM IPTG; (C) 25 μM IPTG; (D) higher-magnification images of the regions highlighted in panel C.

It has been shown that assembly of the biofilm matrix is a checkpoint that controls matrix gene expression (3, 45). More specifically, disruption of matrix assembly leads to an accumulation of cells in the matrix on state (3). Assembly of the biofilm matrix is disrupted in the *degU* mutant strain due to the lack of BslA production (16). Therefore, we wanted to confirm if the increased percentage of matrix on cells observed in the absence of *degU* (Fig. 3F) was a direct consequence of the cells lacking DegU∼P or if it was an indirect consequence of a lack of biofilm matrix. To test this, transcription of *bslA* was induced from a heterologous promoter in the *degU* deletion strain. This generated a genetic background where biofilm formation was restored but where *degU* (and, thus, DegU∼P) was absent (16). In the presence of IPTG, *bslA* was transcribed and biofilm formation was restored (16). However, the balance of matrix on to matrix off cells was not returned to that of the wild-type strain and remained biased toward the matrix on state (Fig. 4A). Moreover, an enhanced colony complexity was exhibited when *bslA* expression was induced in the *degU* strain and not when *bslA* was expressed in either the 3610 wild-type strain or the *bslA* mutant strains (Fig. 4B to D). In combination, these findings are supportive of the higher levels of transcription from the operons needed for synthesis of the biofilm matrix in the *degU* mutant, which manifests as enhanced biofilm formation when BslA is present. We therefore conclude that DegU∼P controls transcription from the *eps* and *tapA* promoter elements in a manner that is not influenced by the lack of biofilm matrix assembly. The implications for DegU simultaneously playing a positive and a negative role in regulating biofilm formation are discussed later.

### The level of DegU∼P controls the probability of cell-activating matrix gene expression.

**FIG 5:**
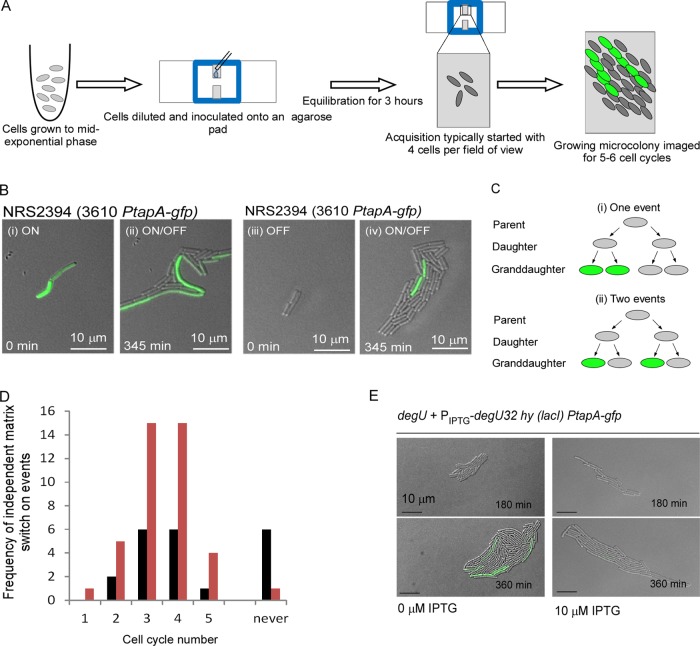
Real-time analysis of matrix gene expression in single cells. (A) Schematic depicting experimental setup. (B) Representative still frames (merged DIC and GFP channel) taken from time-lapse movies for strain NRS2394 (3610 *sacA*::*PtapA-gfp*) demonstrating the two broad mechanisms by which a steady-state bimodal population of matrix on (GFP-positive) and matrix off (GFP-negative) cells can be derived. (i) Matrix on cells grow, divide, and switch off matrix gene expression to generate a mixed population (ii) after 180 min. (iii) Matrix off cells grow, divide, and initiate matrix gene expression to generate a mixed matrix on/off population (iv) after 180 min. (C) Schematic representing how a matrix on event was defined as independent. (D) Histogram showing the distribution of independent matrix on events for the wild-type strain (NRS2394) (black bars) and the *degU* mutant strain (NRS2745) (orange bars) carrying the P*tapA-gfp* reporter fusion over each cell cycle. (E) Representative still frames (DIC and GFP channel) taken from time-lapse movies are presented for strain NRS2759 [*sacA*::*PtapA-gfp degU amyE*::*P*(Hy)-*spank-degU32*(Hy)-*lacI*] in the presence and absence of IPTG, as indicated. Progression from top to bottom is with respect to time, as noted on the micrograph.

To gain an understanding of the molecular mechanism underpinning DegU∼P control of matrix gene expression, we aimed to determine how the increase in the proportion of matrix on cells in the absence of DegU∼P was established. We postulated that it could be the consequence of one of two mechanisms: either matrix on events could occur in the *degU* population at a higher frequency, or alternatively, the matrix on state could be inherited by *degU* daughter cells for more generations, increasing the proportion of matrix on cells over time through the process of division and inheritance. These two possibilities could not be distinguished from our flow cytometry data, where cells from a single time point of biofilm development were examined. Therefore, we employed real-time analysis of matrix gene expression during microcolony development; for a schematic of our experimental setup, see Fig. 5A (41, 46). As anticipated, there were two routes by which a steady-state bimodal *PtapA* expression profile could be established in the mature microcolony. First, a proportion of the cells that started in the matrix on state turned off (e.g., Fig. 5Bi and ii), and second, a proportion of the cells that started in a matrix off state turned on (e.g., Fig. 5Biii and iv; see Movie S1 in the supplemental material).

The total number of independent matrix on events was calculated from multiple wild-type and *degU* microcolonies whose development was monitored over 4 to 5 cell division cycles (for detail and controls, see the text in the supplemental material). Data from a series of movies where a total of 62 wild-type and 62 *degU* mutant matrix off cells represented the starting population were collated. Independent matrix on events were defined as those that were separated from other matrix on events by at least two cell division events (Fig. 5C; see Fig. S3 in the supplemental material for an example). The frequency of independent matrix on events that occurred in each cell cycle was calculated (Fig. 5D). Analysis indicated that there were no differences in the distribution of matrix on events during microcolony development with respect to the cell division cycle between the wild-type and *degU* mutant (Fig. 5D) (*P* < 0.96). However, a higher frequency of matrix on events was recorded for the *degU* mutant than for the wild-type strain (*P* < 0.002) (Fig. 5D). It was noted that for some microcolonies that were tracked, no matrix on events occurred during the analysis period. This happened more often during wild-type microcolony development than during *degU* microcolony development (Fig. 5D). These findings indicate that DegU∼P affects the frequency of activating matrix gene expression in the population.

We next tested if increasing the level of DegU∼P decreased the probability that a cell would activate matrix gene expression. The strain where the *degU32*(Hy) gene is under the control of the IPTG-inducible promoter was used to test this prediction (Fig. 2C). The starter cultures were grown in the absence of IPTG and transferred to imaging conditions, where they were incubated either in the absence of IPTG or in the presence of 10 μM IPTG. Over time, the basal level of transcription of the *degU32*(Hy) gene that occurs in the absence of IPTG was sufficient to establish a bimodal distribution of *PtapA* expression (Fig. 5E) (this mimics a low level of DegU∼P [36]). In contrast, in the presence of 10 μM IPTG and, therefore, significant transcription of the *degU32*(Hy) gene, matrix on cells were far less prevalent (Fig. 5E). As described above, we recorded the number of independent matrix on events over 330 min (4 to 5 cell division cycles). In the absence of IPTG, 12 independent matrix switch on events were observed; in contrast, for the same size of starting population, in the presence of 10 μM IPTG, no independent matrix switch on events were observed (*P* < 0.05). Taken together, these data indicate that increased levels of DegU∼P reduce the frequency of matrix on events to a point where most cells cannot switch on at all.

### DegU∼P influences the level of Spo0A∼P.

**FIG 6:**
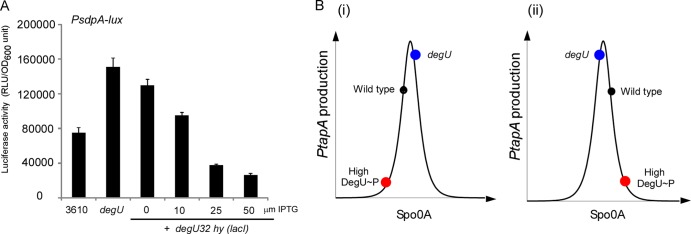
High levels of DegU∼P reduce expression from the *sdpA* promoter. (A) Luciferase activity of cells grown under biofilm formation conditions for 17 h for strains NRS3915 (3610 *sacA*::*PsdpA-lux*), NRS3917 (*degU sacA*::*PsdpA-lux*), and NRS3919 [*degU sacA*::*PsdpA-lux* and *P*(Hy)-*spank-degU32*(Hy)-*lacI*] in the presence and absence of IPTG, as indicated. Averages from 3 experiments are presented, and the error bars represent the standard error of the mean. RLU, relative light units; OD_600_, optical density at 600 nm. (B) Schematic representation of the band-pass response of the *tapA* promoter to changes in the levels of Spo0A∼P. Colored dots, theoretical level of *PtapA* activity in the designated genetic backgrounds; red dots, high levels of DegU∼P; black dots, wild-type levels; blue dots, impact of deleting *degU*. (i) Spo0A∼P levels decreasing with increasing DegU∼P levels; (ii) Spo0A∼P levels increasing with increasing DegU∼P levels. In either case, the *tapA* expression level is low when the level of DegU∼P is high.

We next turned to the question of how DegU∼P controlled the frequency of matrix gene expression. Previous work has shown that matrix gene expression is activated when the level of Spo0A∼P is at a threshold level (33). We therefore proposed that DegU∼P could potentially influence the frequency of matrix gene expression in the population by modulating the level of Spo0A∼P in the cell. This was hypothesized on the basis of the fact that our data demonstrated that DegU∼P does not regulate expression from the matrix gene promoters directly (Fig. 3C to E). To begin to explore the validity of this proposal, we examined the influence of changing the levels of DegU∼P in the cell on transcription of another Spo0A∼P-regulated operon, namely, the *sdpA* operon, which controls the production of the sporulation delay protein. Transcription of the *sdpA* operon has been shown to be highly sensitive to Spo0A∼P levels (47), and it was therefore chosen for use in this study. Analysis of the transcription of the *sdpA* operon allowed us to examine the impact of both increasing and decreasing the level of DegU∼P on an Spo0A∼P-controlled promoter that is controlled in a manner distinct from that in which the complex regulatory circuitry controls matrix gene expression (48), thus allowing us to assess generality. To monitor expression, we used a reporter construct where the *sdpA* promoter region had been fused to the luciferase operon (*PsdpA-lux*) (49). This construct was transduced into the wild-type, *degU* mutant, and *degU32*(Hy) strains (see Table S1 in the supplemental material). To assess luciferase activity, cells were collected from complex colonies and light production was quantified. We observed that activity from the *PsdpA-lux* reporter fusion was 2-fold higher in the *degU* mutant strain than in the wild-type strain (Fig. 6A) (*P* < 0.01). Moreover, activity from the *PsdpA-lux* reporter fusion decreased in the *degU32*(Hy) strain as the level of IPTG (and, so, the level of DegU∼P) was increased. Light production from the *PsdpA-lux* reporter fusion decreased by 2.8-fold compared with the wild-type expression levels when 50 μM IPTG was added to the growth medium (Fig. 5A) (*P* < 0.01). These data are consistent with our hypothesis that changing the level of DegU∼P affects the level of Spo0A∼P and, thus, in this case, transcription from the *PsdpA-lux* reporter fusion.

### High levels of DegU∼P promote sporulation.

**FIG 7:**
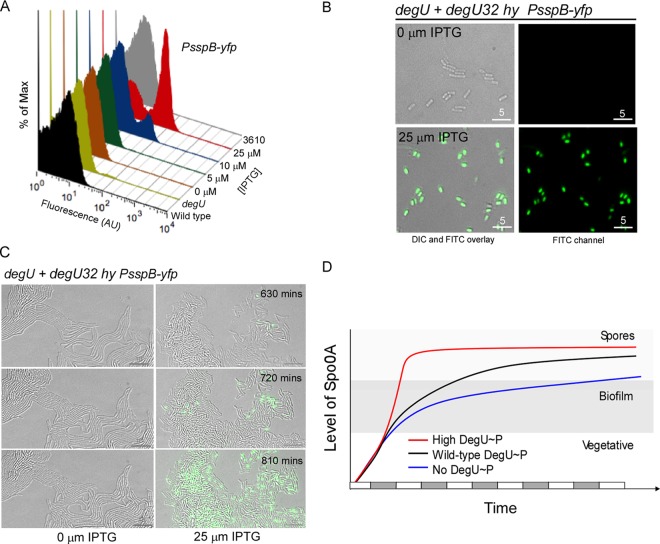
High levels of DegU∼P promote sporulation. (A) Flow cytometry analysis of NRS4269 (3610 *lacA*::*PsspB-yfp*), NRS4265 (*degU lacA*::*PsspB-yfp*), and NRS4266 [*degU P*(Hy)-*spank-degU32*(Hy)-*lacI lacA*::*PsspB-yfp*] cells grown under biofilm formation conditions for 17 h. Cells were grown in the presence or absence of IPTG, as indicated. (B) DIC and fluorescence (YFP) microscopy images of NRS4266 cells analyzed in the experiments whose results are shown in panel A. (C) Representative still frames (DIC and YFP channel) taken from time-lapse movies are presented for strain NRS4266 in the presence and absence of IPTG. The progression from top to bottom is with respect to time. (D) A model predicting the influence of DegU∼P on the level of Spo0A∼P over time. The *x* axis represents time, and the gray and white bars along the *x* axis represent cell division events. Black line, wild type; blue line, *degU* mutant; red line, the presence of high levels of DegU∼P. The level of Spo0A∼P is on the *y* axis, and the theoretical threshold levels of Spo0A∼P needed to activate matrix production and sporulation are indicated.

In combination, the analysis of matrix gene expression and the *PsdpA* transcriptional data presented thus far clearly point to DegU∼P influencing the level of Spo0A∼P in the cell. However, transcription from both the matrix gene operons and the *sdpA* operon is possible only within a band-pass level of Spo0A∼P (Fig. 6B). If the level of Spo0A∼P is below a certain threshold, gene expression is not activated (24, 50). In contrast, at the other end of the scale, if the level of Spo0A∼P increases above a higher threshold, gene expression is inhibited (24, 51). Therefore, the data presented in this study are consistent with high levels of DegU∼P triggering Spo0A∼P to fall either below (Fig. 6Bi) or above (Fig. 6Bii) the Spo0A∼P band pass; i.e., both scenarios would result in low levels of expression from the *epsA*, *tapA*, and *sdpA* promoter elements. Hence, to determine if the level of Spo0A∼P was an increasing or decreasing function of the level of DegU∼P, we analyzed the behavior of a transcriptional fusion to the promoter region for the *sspB* gene (3). The *sspB* gene is transcribed specifically when the level of Spo0A∼P is high and encodes a small acid-soluble protein found in sporulating cells (52). The *PsspB-yfp* transcriptional reporter fusion was transduced into the wild-type, *degU* mutant, and *degU32*(Hy) strains (see Table S1 in the supplemental material). Flow cytometry analysis was used to determine the percentage of the biofilm population that had activated transcription from the *PsspB-yfp* reporter fusion. Additionally, single-cell microscopy analysis was performed to qualitatively confirm the *PsspB* expression profiles calculated by flow cytometry and quantify the percentage of the population that had progressed to form a phase-bright spore. Entirely consistent with previous data (3, 16), analysis revealed that after 17 h of incubation in the biofilm, the wild-type strain exhibited a low level of sporulation, with 1% of the population being defined as *PsspB-yfp* positive and 2% having formed a phase-bright spore (Fig. 7A and data not shown). For the *degU* mutant strain, *PsspB-yfp*-positive cells were detected in 0.02% of the population, and no phase-bright spores were observed in the population that was examined (*n* = 1,000) (Fig. 7A and data not shown). Next, the impact of expressing the *degU32*(Hy) gene on the process of sporulation in the colony biofilm was tested. In the absence of IPTG, 0.1% of the population expressed the *PsspB-yfp* reporter fusion and 0.01% had formed phase-bright spores (Fig. 7A and B and data not shown). This level rose to 9% (YFP positive) and 8% (phase-bright spores) of the population in the presence of 10 μM IPTG (Fig. 7A and B and data not shown), with a further rise to 39% (YFP positive) and 45% (phase-bright spores) of the population occurring in the presence of 25 μM IPTG (Fig. 7A and B and data not shown). A similar increase in *sspB* gene expression upon induction of *degU32*(Hy) expression was also observed during microcolony development, confirming the comparable behavior of the *PsspB* reporter fusion in the two different growth regimes (Fig. 7C). These findings demonstrate that increasing the level of DegU∼P in the cell triggers sporulation and are consistent with data demonstrating that in laboratory isolates of B. subtilis, introduction of the *degU32*(Hy) gene promotes early spore formation under planktonic growth conditions (53).

## DISCUSSION

### DegU∼P controls the probability of a cell becoming a matrix producer.

The response regulator DegU has dual roles in controlling biofilm formation by B. subtilis, functioning as both an activator and an inhibitor at intermediate and high levels, respectively (36). Recent work has focused on the mechanism by which DegU∼P activates biofilm formation through the biosynthesis of BslA (14, 16, 17, 21). Here, we investigated the mechanism by which DegU∼P inhibits biofilm formation. Using both a *degU* deletion strain and a strain where the level of DegU∼P could be finely tuned, we show that DegU∼P inhibits transcription from the *eps* and *tasA* operons (Fig. 2E and F and 3A, B, and F). Until now, a role for DegU∼P in controlling transcription from the *eps* or *tapA* promoter regions has not been identified (e.g., see reference 54). This could perhaps be a reflection of the strains and conditions used for previous analyses, as it is known that wild isolates of B. subtilis (such as the one used in this study) exhibit higher basal levels of transcription from the *eps* and *tapA* loci than laboratory strains (7, 55). The genetic basis for this divergence between the behavior of the wild and domesticated isolates of B. subtilis with respect to biofilm formation has been elucidated (56). Through analysis of microcolony development in real time, we revealed that DegU∼P controls the frequency of matrix on events (Fig. 5D). Subsequent experiments led us to conclude that high levels of DegU∼P result in an increase in the level of Spo0A∼P in the cell, promoting a matrix off, sporulation on state.

### Convergence of the DegU∼P regulatory and Spo0A∼P regulatory pathways.

As noted above, we propose that DegU∼P controls biofilm formation by manipulating the level of Spo0A∼P in the cell. More specifically, we suggest that the level of Spo0A∼P is a function of an increase in DegU∼P. How this is achieved at the molecular level is not yet known, but reasonable hypotheses are that this could be achieved by either increasing the production rate or decreasing the degradation rate of Spo0A∼P. In either case, significant increases in Spo0A∼P net production would have the following consequences: (i) the threshold level required to activate sporulation would be reached after fewer cell cycles, and (ii) the transit time through the matrix on phase would be shortened. This is shown schematically in Fig. 7D. These conjectures are in line both with the established knowledge that there is an inherited cell-autonomous delay in cellular differentiation that occurs before B. subtilis commits to sporulation (57) and also with our findings that high levels of DegU∼P sharply reduce the time to sporulation (Fig. 7A and C). An indirect consequence of early sporulation would be a reduction in the time frame during which productive expression of the genes needed for biofilm formation occurs (Fig. 7D) (51, 57). This fits directly with the block in matrix gene expression shown here in this strain background (Fig. 2E and F, 3A and B, and 5E). At the opposite extreme, when *degU* is deleted and there is no DegU∼P in the system, cells would exhibit a level of Spo0A∼P that would be compatible with matrix gene expression over a time period longer than that for the wild type (Fig. 7D).

### A model for DegU∼P simultaneously functioning as an activator and an inhibitor of biofilm formation.

In the wild-type strain, DegU∼P is needed during biofilm formation to promote the synthesis of BslA (14), which functions alongside the exopolysaccharide and TasA amyloid fibers to facilitate the assembly of the biofilm matrix (16). Thus, the *degU* mutant exhibits a biofilm-minus phenotype. Here, we demonstrate that DegU∼P additionally functions to restrict the proportion of the bacterial community in the biofilm that synthesizes the exopolysaccharide and TasA amyloid fibers and have thus established that DegU∼P has two apparently opposing roles. One can question why B. subtilis has evolved in this way and can postulate that this may be due to the necessity to coordinate the synthesis of BslA, the exopolysaccharide, and TasA to maximize biofilm matrix assembly and to minimize the energy used by each cell of the wild-type strain. A model can be proposed as follows: during the initial stages of biofilm formation, the level of DegU∼P is low, ensuring that the proportion of cells expressing the matrix operons is high (a proposal that is consistent with published data [3]). Production of the exopolysaccharide and TasA amyloid fibers would begin, and over a short period of time, as small changes in the environmental conditions occur, the level of DegU∼P would increase to above the threshold level needed to activate *bslA* transcription. Production of BslA at this point would now allow the biofilm matrix to assemble (16) and would allow the cells to form the BslA-mediated protective barrier (17). Concurrent with or after the activation of *bslA* transcription, at a threshold level of DegU∼P, a decrease in the proportion of cells that transcribe the *eps* and *tasA* operons would occur. Again, this is consistent with data demonstrating that during biofilm development the proportion of matrix on cells decreases over time (3). Assuming a continued increase in the level of DegU∼P during biofilm development, the probability of an individual cell becoming a matrix-producing cell would decrease, and entry to the sporulation pathway would be promoted (Fig. 7D).

### Heterogeneity in the response to DegU∼P in the cell population.

Single-cell techniques allow one to trace the behavior of individuals in a population and the penetrance of a phenotype or trait within the population to be explored (58–60). The impact of DegU within the isogenic B. subtilis population is heterogeneous, as evidenced by only a proportion of the cells being affected by the deletion of *degU* (Fig. 3F). This could result from heterogeneity in the transcription and, therefore, translation of *degU*. However, experimental evidence does not support this. Indeed, it has been demonstrated that *degU* is transcribed in a unimodal manner in laboratory strains (61), and our analysis demonstrates that this is also the case in the strain used for this study (see Fig. S4 in the supplemental material). Furthermore, we have shown here that when the *degU32*(Hy) gene is transcribed from a heterologous promoter that is uniformly expressed in the population, the cells do not exhibit an identical phenotype (Fig. 3A and B). This raises the possibility that levels of DegU∼P (but not those of DegU) vary from cell to cell, something that we have recently investigated mathematically (62). It should be noted that the heterogeneity of DegU∼P activity in the population is not restricted to the control of matrix gene expression described here. In fact, partial penetrance of DegU∼P activity in the total bacterial population has also been observed during flagellum biosynthesis (63). In that study, it was suggested that DegU becomes phosphorylated specifically in nonmotile chaining cells, perhaps in response to completion of the flagellar basal body, and more recent findings have demonstrated that the level of DegU∼P in the cell increases upon arrest of flagellum rotation (64).

### Concluding remarks.

In summary, we have shown that DegU∼P, like Spo0A∼P (33), functions as both an activator and an inhibitor of biofilm formation. The use of regulators with dual function is likely to have evolved to provide a flexible, fast-responding, and perhaps energy-efficient mechanism of control. It is highly probable that other differentiation systems are controlled by similar dual-purpose regulators. Given their central importance, targeting such regulators may provide the key to effective control of many microbial systems in both natural and biotechnological settings.

## Supplementary Material

Supplemental material
