# Supplementary material for: Phosphorylated DegU Manipulates Cell Fate Differentiation in the Bacillus subtilis Biofilm
Source: J Bacteriol. 2014 Jan;196(1):16–27. doi: 10.1128/JB.00930-13 (PMC3911142; doi:10.1128/JB.00930-13)
Supplement: Supplemental material [file supp_196_1_16__index.html]

Supplemental material 

# Phosphorylated DegU Manipulates Cell Fate Differentiation in the Bacillus subtilis Biofilm

## Supplemental material

**Files in this Data Supplement:**

- Supplemental file 1 -

  Supplemental text

  Table S1, strains

  Fig. S1, purified DegS-His6 is capable of phosphorylating DegU-His6*in vitro*, and electrophoretic mobility shift assay with DegU and *aprE* promoter fragment

  Fig. S2, phosphorylated DegU is specifically responsible for modulating expression from the *PtapA* promoter

  Fig. S3, position-independent events occur that activate matrix gene expression during microcolony development

  Fig. S4, transcription from the *degU* promoter is unimodal

  Legend to Movie S1

  PDF, 574K
- Supplemental file 2 -

  Movie S1, growing and differentiating wild-type *B. subtilis* microcolony showing the development of a steady-state bimodal matrix gene expression profile

  MPG, 309K
