# Supplementary material for: Phosphorylated DegU Manipulates Cell Fate Differentiation in the Bacillus subtilis Biofilm
Source: J Bacteriol. 2014 Jan;196(1):16–27. doi: 10.1128/JB.00930-13 (PMC3911142; doi:10.1128/JB.00930-13)
Supplement: Supplemental material [file JB.00930-13_zjb999092944so1.pdf]

Supplemental Text and Figures

DegU~P manipulates cell fate differentiation in the *Bacillus subtilis* biofilm.

Victoria L. Marlow [1], Michael Porter[2], Laura Hobley [1], Taryn B. Kiley [1], Jason R. Swedlow [2], Fordyce A. Davidson [3], Nicola R. Stanley-Wall [1]*

[1] Division of Molecular Microbiology, College of Life Sciences, University of Dundee, Dundee, UK DD1 5EH

[2] Centre for Gene Regulation and Expression, College of Life Sciences, University of Dundee, Dundee, UK DD1 5EH

[3] Division of Mathematics, University of Dundee, Dundee, UK DD1 5EH

Running Title: C*ell fate in the biofilm.*

*To whom correspondence should be addressed:

Nicola R. Stanley-Wall

Division of Molecular Microbiology, College of Life Sciences, University of Dundee, Dundee DD1 5EH.

Tel: +44(0)1382 385136;

Fax: +44(0)1382 388216;

Email: n.r.stanleywall@dundee.ac.uk

Keywords: *Bacillus subtilis*/ biofilm/ DegU/ bistability / sporulation

Strain construction

Plasmid construction

Plasmid pNW105 was constructed to assay *tapA* transcription using LacZ as a reporter. The *tapA* promoter region was amplified from NCIB3610 genomic DNA using primers NSW 50 (5'-TGGCGAATTCATAGACAAATCACACATTGTTTGATCA-3') and NSW 51 (5'-GCCAGAATTCGGATCCATCTTACCTCC TGTAAAACACTGTAA-3'). The amplified fragment was digested with EcoRI and ligated into pDG1633 (1) digested the same. This would allow integration at the non-essential *thrC* locus.

Plasmid pNW108 was constructed to assay *epsA* transcription using LacZ as a reporter. The *epsA* promoter region was amplified from NCIB3610 genomic DNA using primers NSW 52 (5'-TGGAGAATTCTGTACGGCTTGCACTAAATGTACG-3') and NSW 53 (5'-GCCAGAATTCGGATCCATTCATAGCCTTCA GCCTTCCCG-3'). The amplified fragment was digested with EcoRI and ligated into pDG1633 (1) digested the same. This would allow integration at the non-essential *thrC* locus.

Plasmid pNW703 was used to disrupt the *degU* locus; it contained an internal region of DNA from the *degU* coding region and a kanamycin resistance cassette. To construct this plasmid the internal region of *degU* was cut from pBL204 (2) using Bam*HI* and Eco*RI* and ligated into pUC19 (3) also digested Bam*HI* and Eco*RI* creating plasmid pNW701. The kanamycin resistance cassette was cut from plasmid pDG783 (4) using Sph*I* and Xba*I* and ligated into pNW701 also digested with Sph*I* and Xba*I* creating pNW703.

Plasmid pNW704 was constructed to assay *degU* transcription using GFP as a reporter. The plasmid was generated as follows*: PdegU-gfp* (500 bp *degU* promoter region cloned in front of *gfp*) was released from plasmid pNW205 (NSW lab stocks, unpublished), using Eco*RI* and Bam*HI.* The *PdegU-gfp* fragment was then ligated into plasmid into pDR183 (*lacA::erm*) (5) digested the same. This would allow for integration into the non-essential *lacA* locus.

Plasmid pNW730 was constructed to assay *sspB* transcription using YFP production as a reporter. The plasmid was generated as follows: Genomic DNA was prepared from strain HV1049 *amyE::PsspB-yfp* (6) and used as a template to amplify the 240 base pair *sspB* promoter region and *yfp* coding region using NSW 1030 (5' –GCCAGGGCTGCAGGAATTC-3') and NSW1029 (5'-GGTAGCGACCGGCGCTCAGGATCCTTA-3'). The *PsspB-yfp* PCR product was then digested *EcoRI* and *BamHI* and ligated into pDR183 (*lacA::erm*) (5), which would allow for integration into the non-essential *lacA* locus.

Calculation of the frequency of independent Matrix ON events

Starter cultures for analysis were grown in 15% MSgg media prior to dilution and inoculation on the 15% MSgg-agarose pads (see Materials and Methods). After inoculation the cells were allowed to equilibrate for 3 hours prior to image acquisition. At the start of image acquisition all experiments typically started with 4 cells per field of view. Over a period of up to 8.5 hours the cells grew and divided to form a microcolony typically containing between 100-200 cells representing 5-6 cell cycles. As expected (7), the cells in the inoculum were heterogeneous with respect to P*tapA* expression (data not shown) and throughout the real time analysis period the behaviour of the cells diverged. As discussed in the main body of the paper, there were two mechanisms by which an overall steady state, bimodal P*tapA* expression profile was established. First, a proportion of the cells that started in the Matrix ON state turned OFF (e.g. Fig 4Bi & ii) and second, a proportion of the cells that started in a Matrix OFF state turned ON (e.g. Fig. 4B iii & iv & movie S1). An overview of the development and production of GFP fluorescence from the microcolonies formed by the *degU* mutant carrying the P*tapA-gfp* reporter demonstrated that, like the wild type, steady state bimodal P*tapA* expression profiles arose as a consequence of both Matrix ON and Matrix OFF events (data not shown).

We collated data from a series of movies, where a total of 62 wild type and 62 *degU* mutant Matrix OFF cells represented the starting population. The frequency of independent Matrix ON events that occurred in each cell cycle was calculated (Fig 5D). When a Matrix ON event occurred, and was separated from another Matrix ON event by at least two-generations, it was classified as independent and included in the analysis. This is demonstrated by the cell trajectory analysis and the cell genealogy tree presented in Fig. S3. To normalize the data from the different movies we assessed the frequency of the wild type and *degU* mutant strains switching ON matrix gene expression over a defined period of time (330 minutes) that represented between 4-5 cell cycles. Overall, it was observed that the distribution of Matrix ON events with respect to cell cycle was identical for both the wild type and *degU* mutant ($p<0.96$). However, there was a higher frequency of Matrix ON events in the *degU* mutant relative to the wild type strain ($p<0.002$). Moreover, it was also noted that in some experiments, no Matrix ON events occurred and that this happened at a higher frequency in movies of the wild type strain (Fig. 5D). [Note- we established that the two strains grew and replicated at the same rate (data not shown). All experiments typically started with 4 cells in a field of view and ended with ~40-50 cells (data not shown).]

Statistical analysis:

For statistical analysis of the data, the null hypothesis was always set as no difference in the mean measured characteristic for the wild type (WT) and *degU* mutant (D) strain:

H_0 : mu_WT - mu_D = 0

<u>The alternative hypothesis we proposed was that there was a difference.</u>

H_a : mu_WT - mu_D ≠0

To test this:

i) We computed the mean value (mu) for the WT and D data sets.

ii) We next computed the standard deviation (SD) for the WT and D data sets.

iii) We used Excel's Student's –t test with the "test 3" option (small data sets, different standard deviations for test populations) to compute the corresponding P-value)

iv) The null hypothesis was rejected if the P-value was below the threshold P<0.05 and we adopted the alternative hypothesis as our working model. For P-values above this threshold, we concluded that there was no strong evidence to support rejecting the null hypothesis.

The same procedure was also used to compare data sets from the *degUhy 32* strain in the presence and absence of induction.

In summary: No statistically significant difference was observed comparing (i) the mean number of starting cells per field of view during microcolony development, (ii) the mean number of cells per field of view at the end point, (iii) the mean time for a complete cell cycle (in the Matrix OFF state) between the wild type and *degU* mutant or (iv) the mean length (in the Matrix OFF state) of the wild type and *degU* mutant cell prior to division. In conclusion, these data allowed us to reasonably assume that the basic growth characteristics of the wild type strain and *degU* mutant were the same (data not shown).

Table S1 Complete List of Strains used in this study.

Strain	Relevant genotype /Description [a]	Reference, source, or construction[b]
JH642	*trpC2 pheA1*	(8)
168	*trpC2*	BGSC
NCIB3610	Prototroph	BGSC
BAL 2423	JH642*, trpC2 pheA1 sinR*::*cat*	Beth Lazazzera
NRS 1235	JH642, *trpC2 pheA1 sinR::cat*	SPP1 BAL 2423 → JH642
NRS 1287	*3610 amyE::Phy-spank degU32hy-lacI (spc)*	(Verhamme *et al*., 2007)
NRS 1313	*trpC2, pheA, degU::pBL204(cat), amyE::Pspankhy-degU-no P-spec*	(9)
NRS 2052	*3610 thrC*::*PyuaB-lacZ* (*erm*)	(10)
DS1993	3610 *degU::mls*	Daniel Kearns
NRS2226	*3610 thrC*::*PyuaB-lacZ* (*erm*) *degU::pBL204 (cat) amyE::Phy-spank- degU -lacI (spc)*	(10)
NRS2225	*3610 degU::pBL204 (cat) amyE::Phy-spank-degU32hy -lacI (spc) thrC*::*PyuaB-lacZ* (*erm*)	NRS1311→NRS2224
NRS 1302	JH642, *degU::pBL204 (cat)*	(2)
NRS 1311	JH642, *amyE:: Phy-spank- degU32 hy-lacI (spc) degU::pBL204(cat)*	(9)
NRS 1314	3610 *degU*::pBL204 *(cat*)	(9)
NRS 1325	3610 *amyE:: Phy-spank-degUhy 32 (spc) degU::pBL204 (cat)*	(9)
NRS 2241	JH642 *trpC2 pheA1 sacA:PepsA-gfp mut2 (kan)*	(11)
NRS 2242	3610 *sacA:PepsA-gfp mut2 (kan)*	(11)
NRS 2244	3610 *sacA*:*PepsA-gfp mut2 (kan) degU::pBL204(cat)*	SPP1 NRS2241 →NRS1314

Strain	Relevant genotype /Description [a]	Reference, source, or construction[b]
NRS 2245	3610 *sacA:PepsA-gfp mut2 (kan) degU::pBL204(cat) amyE::Pspank-hy- degUhy 32-lacI (spec)*	SPP1 NRS2241→NRS1325
NRS2297	*amyE:Phy-spank-bslA-lacI (spc)*	(12)
NRS2298	*degU::pBL204 (cat) amyE:Phy-spank-bslA-lacI (spc)*	(12)
NRS2299	*bslA*::(*cat*) *amyE:Phy-spank-bslA-lacI (spc)*	(12)
NRS 2394	3610 *sacA*::*PtapA-gfp mut2* (*kan*)	(11)
NRS 2761	3610 *degU::pBL204 (cat) amyE::Phy-spank-degUD56N -lacI (spc) sacA*::*PtapA-gfp mut2* (*kan*)	SSP1 NRS1313→NRS2745
NRS 2745	3610 *sacA*::*PtapA-gfp mut2* (*kan*)*degU::pBL204 (cat)*	SPP1 NRS1302 →NRS2394
NRS 2759	3610 *sacA*::*PtapA-gfp mut2* (*kan*) *degU::pBL204 (cat) amyE:: Phy-spank- degUhy 32-lacI (spc)*	SPP1 NRS1311 →NRS2745
NRS 3067	3610 *sacA*::*PtapA-gfp mut2* (*kan*) *degU::pBL204 (cat) amyE:Phy-spank-bslA-lacI (spc)*	SPP1 NRS2745 → NRS2388
NRS 3374	*168, degU::pNW703(kan)*	pNW703 →3610
NRS 3420	3610, *sacA::PtapA-gfp, sinR::cat*	SPP1 NRS1235 → 2394
NRS 3421	3610, *sacA::PtapA-gfp, sinR::cat, degU::mls*	SPP1 NRS2074 → 3420
NRS 3424	3610, *sacA::PtapA-gfp, sinR::cat, degU::mls amyE:: Phy-spank- degUhy 32-lacI (spc)*	SPP1 NRS 1287→3421
NRS 3388	168, *lacA::PdegU-gfp*	pNW704→ 168
NRS 3389	3610, *lacA::PdegU-gfp*	SPP1 NRS 3389 →3610
NRS 3390	3610, *lacA::PdegU-gfp, degU::kan*	SPP1 NRS 3374 →3389
NRS 3393	3610*, lacA::PdegU-gfp, degU::kan, amyE::degU32 hy (lacI)*	SPP1 NRS 1287→ 3390
CY136	3610, *sacA:: PsdpA-lux (cat)*	(13)

Strain	Relevant genotype /Description [a]	Reference, source, or construction[b]
NRS 3915	3610, *sacA:: PsdpA-lux (cat)*	SSP1 CY136 → 3610
NRS 3917	3610, *sacA:: PsdpA-lux (cat), degU::kan*	SPP1 3374 → 3915
NRS 3919	3610, *sacA:: PsdpA-lux (cat), degU::kan amyE::degU32 hy*	SPP1 1287→ 3917
NRS 4264	*168, lacA::PsspB-yfp (mls)*	pNW730 → 168
NRS 4265	*lacA::PsspB-yfp (mls)*	SPP1 4265 → 3610
NRS 4266	*lacA::PsspB-yfp (mls) degU::pBL204 (cat)*	SPP1 4265 → 1314
NRS 4269	*lacA::PsspB-yfp (mls) degU::pBL204 (cat) amyE::Phy-spank-degU32hy-lacI (spc)*	SPP1 4266 → 1325
NRS 1503	*thrC::PtapA-lacZ (erm)*	pNW105→ JH642
NRS 1515	*thrC::PtapA-lacZ (erm) degU::pBL204 (cat) amyE::Phy-spank- degUD56N -lacI (spc)*	SSP1 1503 →1325
NRS 1529	*thrC::PepsA-lacZ (erm)*	pNW108 → JH642
NRS 1553	*thrC::PepsA-lacZ (erm) degU::pBL204 (cat) amyE::Phy-spank- degUD56N -lacI (spc)*	SSP1 1529 →1325

a. Drug resistance cassettes are indicated as follows: *cat*, chloramphenicol resistance; *kan*, kanamycin resistance; *tet*, tetracycline resistance; and *spc*, spectinomycin resistance.

b. The direction of strain construction is indicated with DNA or phage (SPP1) (→) recipient strain. BGSC represents the *Bacillus* genetic stock centre

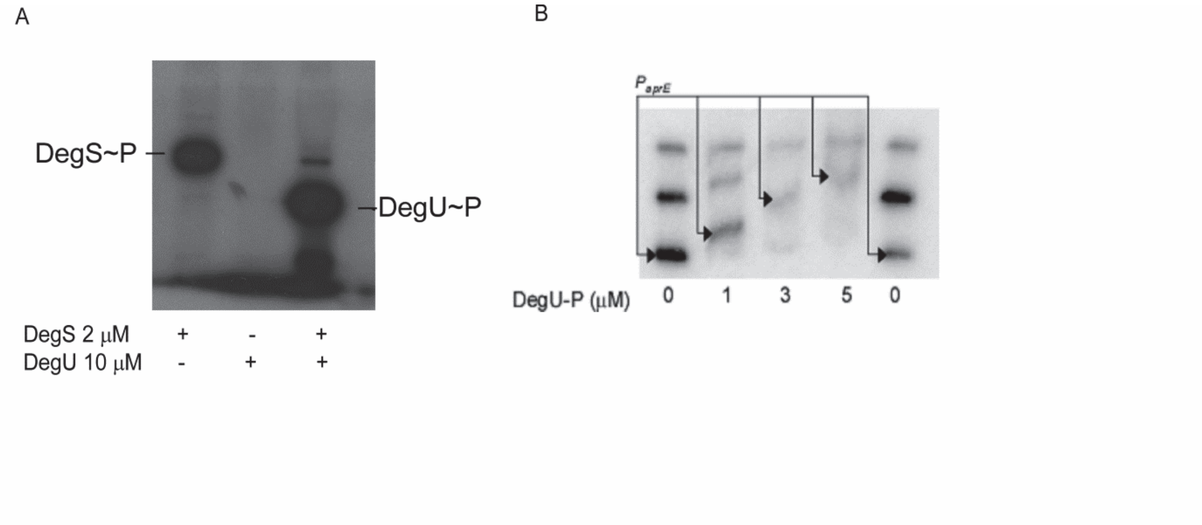

Figure S1: Purified DegS-$_{His6}$ is capable of phosphorylating DegU-$_{His6}$ *in vitro* and Electrophoretic mobility shift assay with DegU and *aprE* promoter fragment. **(A)** Purified DegU-$_{His6}$ and DegS-$_{His6}$ were incubated with ^{32}P γ-ATP, separately and in combination. 1µg of each protein, as required, was added to each reaction mixture. Proteins were mixed with SDS loading buffer before they were loaded onto a 12% SDS polyacrylamide and the gel was dried and exposed to X-ray film for 20 hours at -70°C. Phosphorylated DegS and DegU are indicated. **(B)** DNA binding reactions were conducted with γ-^{32}P labelled DNA corresponding to the region -620 to +24bp surrounding the start of *aprE*. 1 ng labelled DNA was loaded into each lane (after gel extraction), with increasing amounts of purified DegU~P-$_{His6}$. Arrows indicate labelled DNA corresponding to the shifted promoter region of *aprE*.

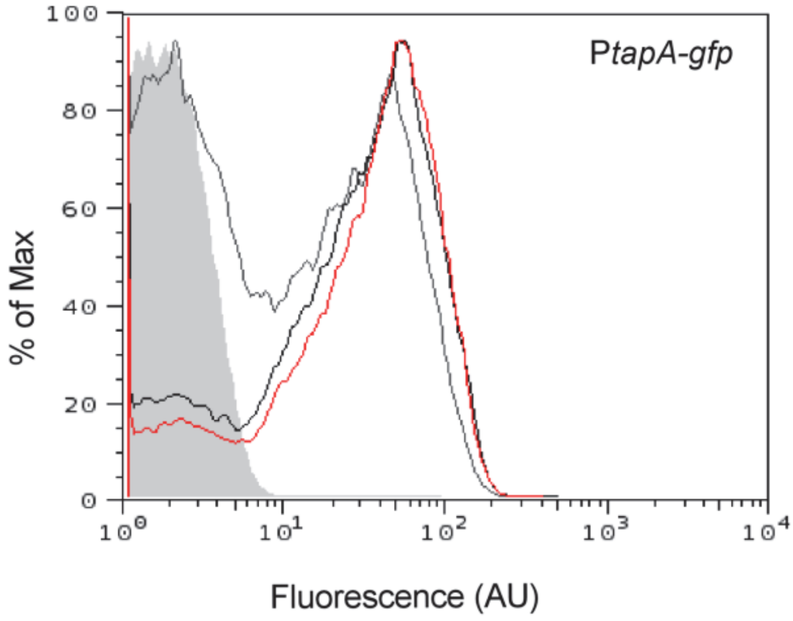

Figure S2: Phosphorylated DegU is specifically responsible for modulating expression from the *PtapA* promoter. Flow cytometry analysis of cells grown under biofilm formation conditions for 17 hours for strains NRS2394 (3610 *sacA::PtapA-gfp*) grey line and NRS2761 (*sacA::PtapA-gfp degU Phy-spank-degUD56N* (*lacI*) with no IPTG, black line and 100 μm IPTG, red line. Strain 3610 provides a non-fluorescent control (Solid grey peak). Fluorescence is in the x-axis in arbitrary fluorescent units. Dataset is representative of the trends observed in three independent experiments.

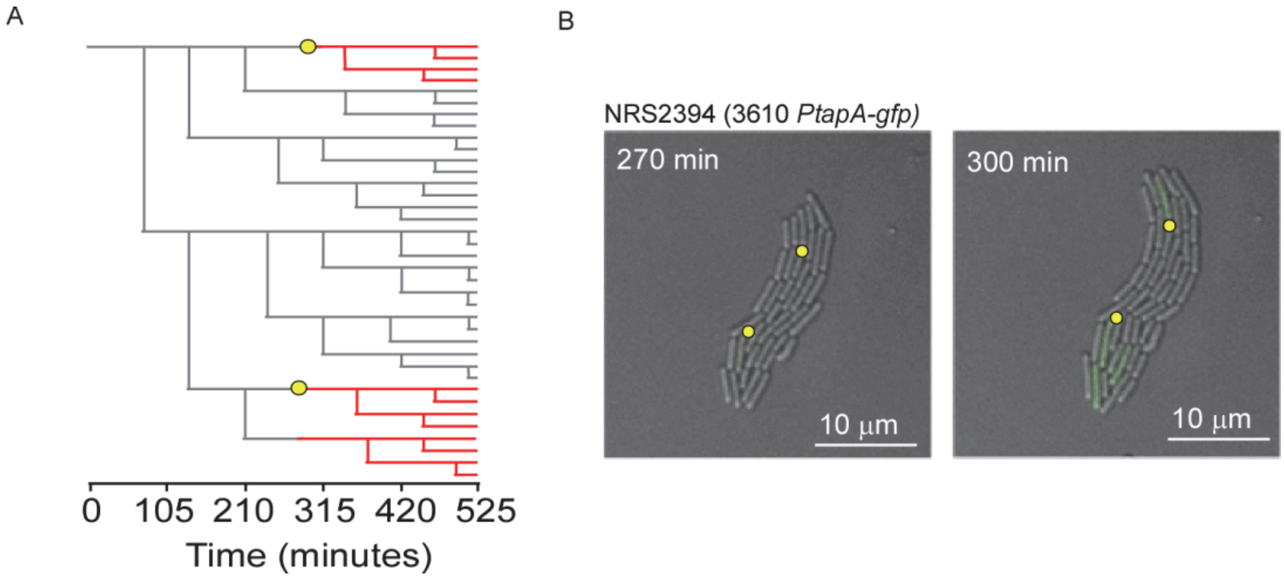

Figure S3 Position-independent events occur that activate matrix gene expression during micrcolony development. (**A**). Cell genealogy tree. An example of a trajectory of a single cell and its daughters over time during microcolony development is presented for strain NRS2394 (3610 *sacA::PtapA-gfp*). The nodes represent the time of division relative to the scale in minutes. Grey lines represent a Matrix OFF state and red lines represent a Matrix ON state. The yellow dots show two independent Matrix ON events and are for reference to part (**B**). (**B**) Representative still frames (DIC and GFP Channel), representing the position of cells that become matrix producers just before (left) and just after (right). In this example the two Matrix ON cells are separated by two generations and are located at distal ends of the microcolony.

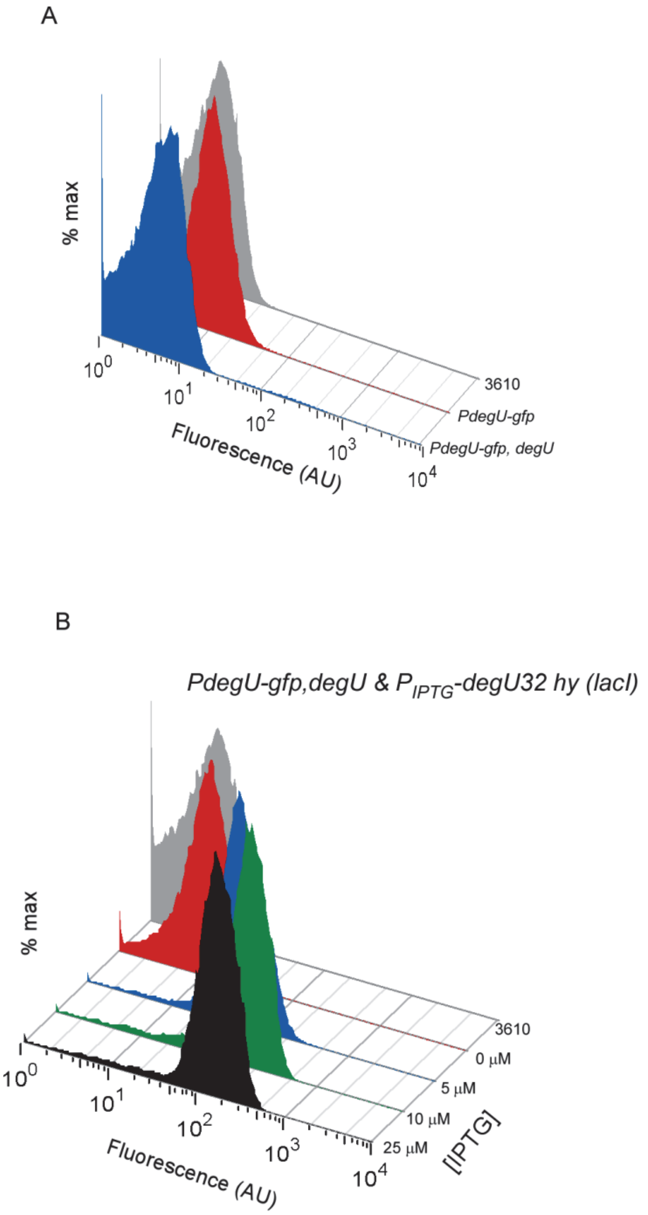

Figure S4: Transcription from the *degU* promoter is unimodal. . Flow cytometry analysis of cells grown under biofilm formation conditions for 17 hours for strains (**A**) NRS3389 (3610 *lacA- PdegU-gfp*) in red, NRS3390 (*lacA- PdegU-gfp degU*, in blue. Strain 3610 provides a non-fluorescent control (Solid grey peak). **(B)** NRS3393 (*3610 lacA- PdegU-gfp degU* P_{IPTG}*-degU-hy* (*lacI*) in the absence (red) and presence of IPTG as indicated (blue, green and black). Strain 3610 provides a non-fluorescent control (Solid grey peak). Fluorescence is in the x-axis in arbitrary fluorescent units. Dataset is representative of the trends observed in three independent experiments.

Movie S1: Growing and differentiating wild type (NRS2394) *B. subtilis* microcolony showing the development of a steady state bimodal matrix gene expression profile. The movie shows 24 frames (6 hours) of the growth of a microcolony harbouring the P*tapA-gfp* construct condensed into 9 seconds. For all frames images were acquired every 15 minutes. Scale bar is in µm.

Supplemental References.

1. **Guerout-Fleury AM, Frandsen N, Stragier P.** 1996. Plasmids for ectopic integration in *Bacillus subtilis*. Gene **180:**57-61.
2. **Stanley NR, Lazazzera BA.** 2005. Defining the genetic differences between wild and domestic strains of *Bacillus subtilis* that affect poly-gamma-dl-glutamic acid production and biofilm formation. Mol Microbiol **57:**1143-1158.
3. **Vieira J, Messing J.** 1982. The pUC plasmids, an M13mp7-derived system for insertion mutagenesis and sequencing with synthetic universal primers. Gene **19:**259-268.
4. **Guerout-Fleury AM, Shazand K, Frandsen N, Stragier P.** 1995. Antibiotic-resistance cassettes for *Bacillus subtilis*. Gene **167:**335-336.
5. **Doan T, Marquis KA, Rudner DZ.** 2005. Subcellular localization of a sporulation membrane protein is achieved through a network of interactions along and across the septum. Molecular Microbiology **55:**1767-1781.
6. **Vlamakis H, Aguilar C, Losick R, Kolter R.** 2008. Control of cell fate by the formation of an architecturally complex bacterial community. Genes Dev **22:**945-953.
7. **Chai Y, Chu F, Kolter R, Losick R.** 2008. Bistability and biofilm formation in *Bacillus subtilis*. Mol Microbiol **67:**254-263.
8. **Perego M, Hoch JA.** 1988. Sequence analysis and regulation of the *hpr* locus, a regulatory gene for protease production and sporulation in *Bacillus subtilis*. J Bacteriol **170:**2560-2567.
9. **Verhamme DT, Kiley TB, Stanley-Wall NR.** 2007. DegU co-ordinates multicellular behaviour exhibited by *Bacillus subtilis*. Mol Microbiol **65:**554-568.
10. **Verhamme DT, Murray EJ, Stanley-Wall NR.** 2009. DegU and Spo0A jointly control transcription of two loci required for complex colony development by *Bacillus subtilis*. J Bacteriol **191:**100-108.
11. **Murray EJ, Strauch MA, Stanley-Wall NR.** 2009. σX is involved in controlling *Bacillus subtilis* biofilm architecture through the AbrB homologue Abh. J Bacteriol **191:**6822-6832.
12. **Ostrowski A, Mehert A, Prescott A, Kiley TB, Stanley-Wall NR.** 2011. YuaB functions synergistically with the exopolysaccharide and TasA amyloid fibers to allow biofilm formation by *Bacillus subtilis*. J Bacteriol **193:**4821-4831.
13. **Chen Y, Cao S, Chai Y, Clardy J, Kolter R, Guo JH, Losick R.** 2012. A *Bacillus subtilis* sensor kinase involved in triggering biofilm formation on the roots of tomato plants. Molecular Microbiology **85:**418-430.
